# An FPGA implementation of Bayesian inference with spiking neural networks

**DOI:** 10.3389/fnins.2023.1291051

**Published:** 2024-01-05

**Authors:** Haoran Li, Bo Wan, Ying Fang, Qifeng Li, Jian K. Liu, Lingling An

**Affiliations:** ^1^Guangzhou Institute of Technology, Xidian University, Guangzhou, China; ^2^School of Computer Science and Technology, Xidian University, Xi'an, China; ^3^Key Laboratory of Smart Human Computer Interaction and Wearable Technology of Shaanxi Province, Xi'an, China; ^4^College of Computer and Cyber Security, Fujian Normal University, Fuzhou, China; ^5^Digital Fujian Internet-of-Thing Laboratory of Environmental Monitoring, Fujian Normal University, Fuzhou, China; ^6^Research Center of Information Technology, Beijing Academy of Agriculture and Forestry Sciences, National Engineering Research Center for Information Technology in Agriculture, Beijing, China; ^7^School of Computer Science, University of Birmingham, Birmingham, United Kingdom

**Keywords:** spiking neural networks, probabilistic graphical models, Bayesian inference, importance sampling, FPGA

## Abstract

Spiking neural networks (SNNs), as brain-inspired neural network models based on spikes, have the advantage of processing information with low complexity and efficient energy consumption. Currently, there is a growing trend to design hardware accelerators for dedicated SNNs to overcome the limitation of running under the traditional von Neumann architecture. Probabilistic sampling is an effective modeling approach for implementing SNNs to simulate the brain to achieve Bayesian inference. However, sampling consumes considerable time. It is highly demanding for specific hardware implementation of SNN sampling models to accelerate inference operations. Hereby, we design a hardware accelerator based on FPGA to speed up the execution of SNN algorithms by parallelization. We use streaming pipelining and array partitioning operations to achieve model operation acceleration with the least possible resource consumption, and combine the Python productivity for Zynq (PYNQ) framework to implement the model migration to the FPGA while increasing the speed of model operations. We verify the functionality and performance of the hardware architecture on the Xilinx Zynq ZCU104. The experimental results show that the hardware accelerator of the SNN sampling model proposed can significantly improve the computing speed while ensuring the accuracy of inference. In addition, Bayesian inference for spiking neural networks through the PYNQ framework can fully optimize the high performance and low power consumption of FPGAs in embedded applications. Taken together, our proposed FPGA implementation of Bayesian inference with SNNs has great potential for a wide range of applications, it can be ideal for implementing complex probabilistic model inference in embedded systems.

## 1 Introduction

Neuroscience research plays an increasingly important role in accelerating and inspiring the development of artificial intelligence (Demis et al., 2017; Zador et al., 2022). Spikes are the fundamental information units in the neural systems of the brain (Bialek et al., 1999; Yu et al., 2020), which also play an important role in information transcoding and representation in artificial systems (Zhang et al., 2020; Gallego et al., 2022; Xu et al., 2022). Spiking neural networks (SNNs) utilize spikes as brain-inspired models are proposed as a new generation of computational framework (Maass, 1997). SNNs have received extensive attention and can utilize many properties of artificial neural networks for deep learning in various tasks (Kim et al., 2018; Shen et al., 2021; Yang et al., 2022).

Numerous neuroscience experiments (Ernst and Banks, 2002; Körding and Wolpert, 2004) have shown that the cognitive and perceptual processes of the brain can be expressed as a probabilistic reasoning process based on Bayesian reasoning. From the macroscopic perspective, Bayesian models have explained how the brain processes uncertain information and have been successfully applied in various fields of brain science (Shi et al., 2013; Chandrasekaran, 2017; Alais and Burr, 2019). In contrast, recent studies focus on implementing SNNs using probabilistic graphical models (PGMs) at the micro level (Yu et al., 2018a,b, 2019; Fang et al., 2019). However, the realization of PGMs is considerably slow due to the sampling process. Since probabilistic sampling on SNNs involves massive probabilistic computations that can consume a lot of time and many computationally intensive operations are involved in processing the data in the neural network, the inference speed will be even slower with the scale of the problem. In some practical application scenarios such as medical diagnosis, environmental monitoring, intelligent monitoring, etc., these problems lead to poor real-time application, which causes a series of problems. Therefore, we want to do some acceleration and improvements to meet the demand for speed in real applications. At present, there are dedicated hardware designs for SNNs (Cai et al., 2018; Liu et al., 2019; Fang et al., 2020; Han et al., 2020; Zhu et al., 2022), and for PGMs based on conventional artificial neural networks (Cai et al., 2018; Liu et al., 2020; Fan et al., 2021; Ferianc et al., 2021). Yet, there are few studies for hardware platforms to implement PGM-based SNNs. Therefore, it is highly demanding and meaningful for hardware acceleration of PGM-based SNNs, not only for simulation speed-up but for neuromorphic computing implementation (Christensen et al., 2022).

In this study, we address this question by utilizing FPGA hardware to implement a recently developed PGM-badsed SNN model, named the sampling-tree model (STM) (Yu et al., 2019). The STM is an implementation of spiking neural circuits for Bayesian inference using importance sampling. In particular, The STM is a typical probabilistic graphical model based on a hierarchical tree structure with a deep hierarchical structure of layer-on-layer iteration and uses a multi-sampling mode based on sampling coupled with population probability coding. Each node in the model contains a large number of spiking neurons that represent samples. The STM process information based on spikes, where spiking neurons integrate input spikes over time and fire a spike when their membrane potential crosses a threshold. With these properties, the STM is a typical example of PGM-based SNN for Bayesian inference. The software implementation of sampling-based SNN is very time-consuming, and actual tasks are limited by the model running speed on CPU. Therefore, to fulfill our requirements for the running speed of the model, it is necessary to choose a hardware platform for designing a hardware accelerator.

Here we need to consider which hardware platform is chosen to better implement the design of the accelerator.

*ASIC-based design implementations:* Compared with general integrated circuits, ASIC has the advantages of smaller size, lower power consumption, improved reliability, improved performance,and enhanced confidentiality. ASICs can also reduce costs compared to general-purpose integrated circuits in mass production. Ma et al. (2017) designed a highly-configurable neuromorphic hardware coprocessor based on SNN implemented with digital logic, called Darwin neural processing unit (NPU), which was fabricated as ASIC in SMIS's 180 nm process for resource-constrained embedded scenarios. Tung et al. (2023) proposed a design scheme for a spiking neural network ASIC chip and developed a built-in-self-calibration (BSIC) architecture based on the chip to realize the network to perform high-precision inference under a specified range of process parameter variations. Wang et al. (2023) proposed an ASIC learning engine consisting of a memristor and an analog computing module for implementing trace-based online learning in a spiking neural network, which significantly reduces energy consumption compared to existing ASIC products of the same type. However, ASIC requires a long development cycle and is risky. Once there is a problem, the whole piece will be discarded. Consequently, we do not consider the use of ASIC for design here.

*FPGA-based design implementations:* FPGA has a shorter development cycle compared to ASIC, is flexible in use, can be used repeatedly, and has abundant resources.

Ferianc et al. (2021) proposed an FPGA-based hardware design to accelerate Bayesian recurrent neural networks (RNNs), it can achieve up to 10 times speedup compared with GPU implementation. Wang (2022) implemented a hardware accelerator on FPGA for the training and inference process of Bayesian belief propagation neural network (BCPNN), and the computing speed of the accelerator can improve the CPU counterpart by two orders of magnitude. However, RNN and BCPNN in the above two designs are essentially traditional neural network architectures, which are different from the hardware implementation of the SNN architecture and cannot be directly applied to our SNN implementation.

In addition, Fan et al. (2021) proposed a novel FPGA-based hardware architecture to accelerate BNNs inferred through Monte Carlo, it can achieve up to nine times better compute efficiency compared with other state-of-the-art BNN accelerators. Awano and Hashimoto (2023) proposed a Bayesian neural network hardware accumulator called B2N2, i.e., Bernoulli random number-based Bayesian neural network accumulator, which reduces resource consumption by 50% compared to the same type of FPGA implementation. For the above two designs, the hardware architecture proposed by Fan and Awano cannot be used for the acceleration of the STM, because the variational inference model and the Monte Carlo inference model are not suitable for importance sampling, but STM needs to be sampled through importance sampling. In other words, the hardware architecture is different due to the different models, so we cannot use these two hardware architectures to accelerate STM on the FPGA.

In summary, many previous designs were implemented on FPGAs because ASIC is less flexible and complex than FPGAs (Ju et al., 2020). GPUs often perform very well on applications that benefit from parallelism, and are currently the most widely used platform for implementing neural networks. However, GPUs are not able to handle spike communication well in real-time, while the high energy consumption of GPUs leads to limitations in some embedded scenarios. Therefore, we chose the FPGA as a compromise solution, which provides reasonable cost, low power consumption, and flexibility for our design. Furthermore, for some FPGA-based design implementations, due to the limitations of the traditional ANN neural network architecture (Que et al., 2022) and some inference models are not suitable for sampling (Fan et al., 2022), we also need to design a hardware implementation suitable for importance sampling (Shi and Griffiths, 2009). Based on the above design reference and our previous work that the STM of a neural network model for Bayesian inference, we finally chose FPGA to complete the design of the STM accelerator, and also complete the neural network model construction of Bayesian inference on FPGA with the help of PYNQ framework to achieve the acceleration of STM. The overall design idea is as follows. Firstly, optimize the model inference part of the algorithm to make full use of FPGA resources to improve program parallelism, thus reducing the computing delay, and complete the design of custom hardware IP cores. Secondly, the designed IP core is connected to the whole hardware system, and the overall hardware module control is realized according to the preset algorithm flow through the PYNQ framework.

The main contributions of this work are as follows:

- We are the first work targeting acceleration of STM on the FPGA board, and the inference results of the STM implemented on the FPGA are similar to the inference results implemented by the CPU;
- We implemented the acceleration of the STM on a Xilinx Zynq ZCU104 FPGA board, and we also found that the acceleration on the FPGA increases with the problem size, such as the number of model layers, the number of neurons, and other factors;
- We demonstrate that the neural circuits we implemented on the FPGA board can be used to solve practical cognitive problems, such as the integration of multisensory, it can also efficiently perform complex Bayesian reasoning tasks in embedded scenarios.

## 2 Related work

### 2.1 Bayesian inference with importance sampling

Existing neural networks using variational-based inference methods such as belief propagation (BP) (Yedidia et al., 2005) and Monte Carlo (MC) (Nagata and Watanabe, 2008) can obtain accurate inference results in some Bayesian models. However, most Bayesian models in the real world are more complex. When using BP (George and Hawkins, 2009) or MCMC (Buesing et al., 2011) to implement Bayesian model inference, each or each group of neurons generally has to implement a different and complex computation in these neural networks. In addition, since spiking neural networks require multiple iterations to obtain optimal Bayesian inference results, they are more complicated to implement. Therefore, STM employs the tree structure of Bayesian networks to convert global inference into local inference through network decomposition. Importance sampling is introduced to perform local inference, which ensures that each group of neurons works simply, making the model suitable for large-scale distributed computing.

Unlike the traditional method of sampling from a distribution of interest, we use importance sampling to implement Bayesian inference for spiking neural networks, which is a method of sampling from a simple distribution to achieve the estimation of a certain function value. When given the variable *y*, the conditional expectation of a function *f*(*x*) is estimated by importance sampling as:


(1) $$\begin{array}{l} E(f(x)|y)=\sum_{x}f(x)P(x|y)=\frac{\sum_{x}f(x)P(y|x)P(x)}{\sum_{x}P(y|x)P(x)}\\ \;=\frac{E{\left(f(x)P(y|x)\right)}_{P(x)}}{E{\left(P(y|x)\right)}_{P(x)}}\approx \sum_{x^{i}}f(x^{i})\frac{P(y|x^{i})}{\sum_{x^{i}}P(y|x^{i})},x^{i}\sim P(x). \end{array}$$

where *x*^*i*^ follows the distribution *P*(*x*). This equation transforms the conditional expectation *E*(*f*(*x*)|*y*) into a weighted combination of normalized conditional probabilities $P\left(y|x^{i}\right)/\underset{x^{i}}{\sum }P\left(y|x^{i}\right)$. Importance sampling can be used to draw a large number of samples from a simple prior, and skillfully convert the posterior distribution into the ratio of likelihood, thereby estimating the expectation of the posterior distribution.

### 2.2 Sampling-tree model with spiking neural network

**Figure 1:**
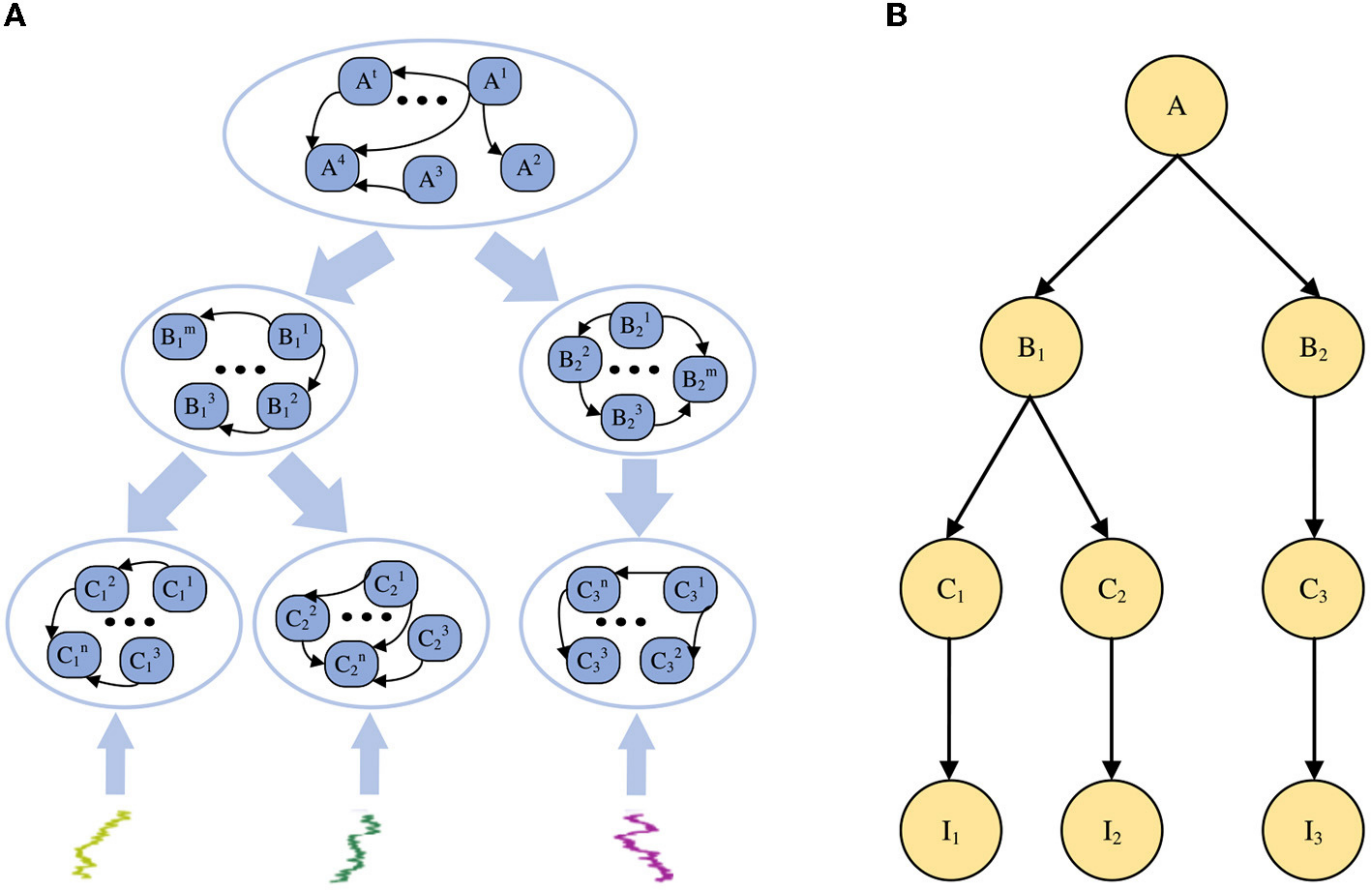
Sampling-tree model. **(A)** An example of the STM in spiking neural networks. **(B)** A tree-structured Bayesian network corresponding to the STM in **(A)**.

To build a general-purpose neural network for large-scale Bayesian models, the STM was proposed in the previous work (Yu et al., 2019), as shown in Figure 1. As a spiking neural network model for Bayesian inference, STM is also a probabilistic graph model with an overall hierarchical structure. Each node in the graph has a large number of neurons as sample data.

The STM is used to explain how Bayesian inference algorithms can be implemented through neural networks in the brain, building large-scale Bayesian models for SNN. In contrast to other Bayesian inference methods, the STM focuses on multiple sets of neurons to achieve probabilistic inference in PGM with multiple nodes and edges. Performing neural sampling on deep tree-structured neural circuits can transform global inference problems into local inference tasks and achieve approximate inference. Furthermore, since the STM does not have neural circuits specifically designed for a specific task, it can be generalized to solve other inference problems. In summary, the STM is a general neural network model that can be used for distributed large-scale Bayesian inference.

In this model, the root node of the Bayesian network is the problem or reason that needs to be inferred in our experiment, the leaf node represents the information or evidence we receive from the outside world, and the branch nodes are the intermediate variable of the reasoning problem. From the macroscopic perspective, the STM is a probabilistic graphical model with a hierarchical tree structure. From the neuron level, each node in the model contains a group of spiking neurons, and multiple connections between these neurons. Each spiking neuron is regarded as a sample from a special distribution, and the information transmission or probability calculation in the model is achieved through the connections between neurons.

### 2.3 Hardware implementation using PYNQ framework

**Figure 2:**
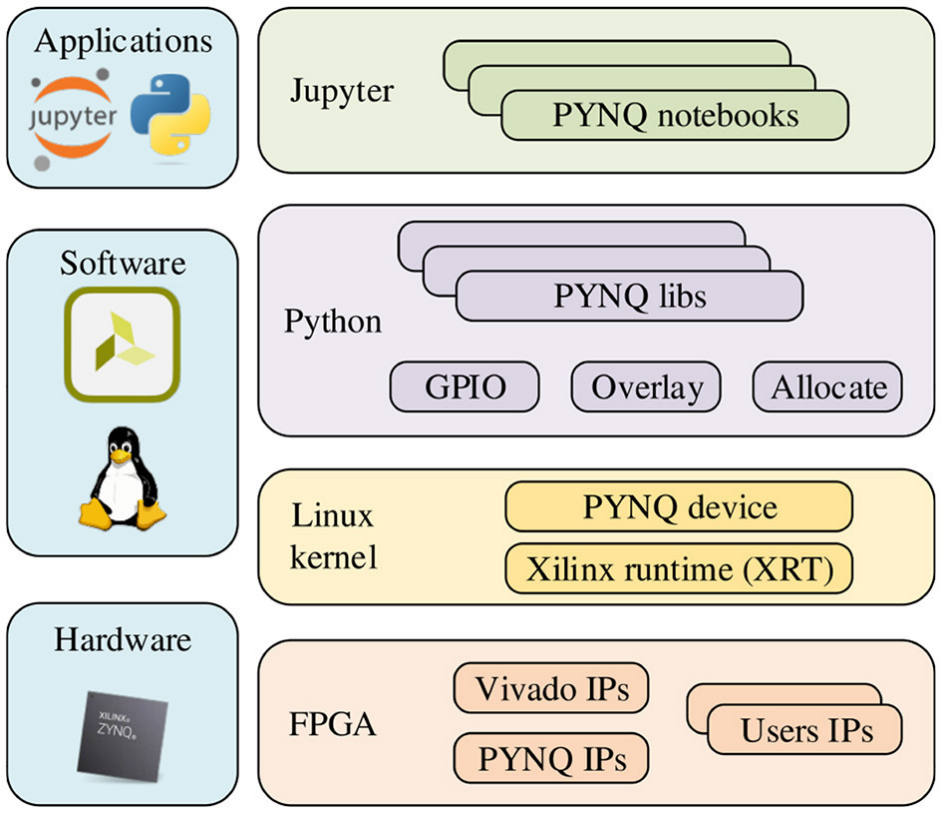
Overall framework of using PYNQ to develop Zynq.

PYNQ provides a Jupyter-based framework and Python API for designing programmable logic circuits using the Xilinx adaptive computing platform instead of using ASIC-style design tools. PYNQ consists of three layers: application layer, software layer, and hardware layer. The overall framework is shown in Figure 2. There have been many works implementing neural network acceleration on FPGAs with the help of the PYNQ framework before this.

Tzanos et al. (2019) implemented the acceleration of the Naive Bayesian neural network algorithm on the Xilinx PYNQ-Z1 board. The hardware accelerator was evaluated on Naive Bayes-based machine learning applications. Ju et al. (2020) proposed a hardware architecture to enable efficient implementation of SNNs and validate it on the Xilinx ZCU102. However, this design directly mapped each different computing stage to a hardware layer. Although this approach can improve the parallelism of the program, this direct mapping method would consume a great deal of the hardware resources or even exceed them. Awano and Hashimoto (2020) proposed an efficient inference algorithm for BNN, named BYNQNet, and its FPGA implementation. The Monte Carlo inference method that this design was based on belongs to variational inference, which is very complicated in implementing larger-scale impulsive neural network models, and the Monte Carlo inference method is not suitable for sampling models.

In our work, we focus on ensuring the inference accuracy of the STM on FPGAs while improving performance. Since the PYNQ framework provides a Python environment that integrates hardware Overlay for easy porting. And with the PYNQ framework, we can implement hardware execution in parallel while creating high-performance embedded applications, and execute more complex analysis algorithms through Python programs, the performance of which can be close to desktop workstations. It also has the advantages of high integration, small size, and low power consumption. When using the PYNQ framework, the tight coupling between PS (Processing System, i.e., ARM processor) and PL (Programmable Logic, i.e. FPGA part) can achieve better responsiveness, higher reconfigurability, and richer interface functions than traditional methods. The simplicity and efficiency of the Python language and the acceleration provided by programmable logic are also fully utilized. Finally, Xilinx has simplified and improved the design of Zynq-based products on the PYNQ framework by combining a hybrid library that implements acceleration within Python and programmable logic. This is a significant advantage over traditional SoC approaches that cannot use programmable logic. Therefore, we implement the Bayesian neural network inference algorithm on Xilinx ZCU104 with the help of the PYNQ framework.

## 3 System analysis

In this section, we first summarize the basis of our work on implementing probabilistic inference algorithms for the brain through neural networks. We then analyze the difficulties of accelerating the probabilistic inference algorithm for running neural network models and briefly describe how we address these difficulties.

### 3.1 Neural network implementation

**Figure 3:**
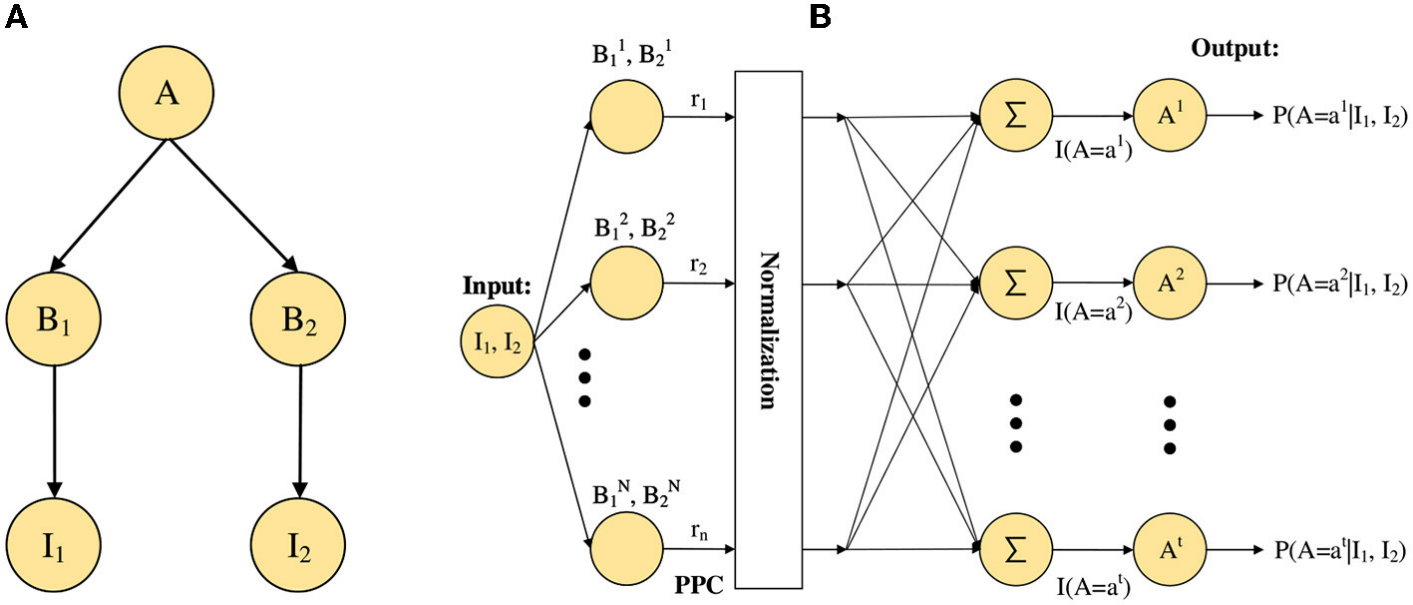
The example of Bayesian network. **(A)** A simple Bayesian neural network model. **(B)** The neural network architecture of the STM for the basic network as in **(A)**.

In this subsection, we take the neural network shown in Figure 3A as an example, and we consider the following two aspects in the implementation of the neural network: First, for the stimulus encoding problem, it is important to know how to accomplish the activities of neurons from stimulus input. Second, for the estimation of posterior probability, it is also necessary to consider how the activities of neurons realize the estimation of posterior probability because our final inference result requires the expectation over posterior distribution.

For the first problem, we convert stimulus input information into the activities of neurons through probabilistic population codes (PPCs) (Ma et al., 2006, 2014). According to PPCs, the activities of these neurons encoding stimuli inputs, *I*_1_, *I*_2_, and others, can be obtained neuronal activity of the root node *A*. For the second problem, we divide it into two steps, one is the calculation of the posterior probability, and the other is the neural implementation of the posterior probability. Based on importance sampling, we can estimate the posterior probability by the ratio approximation of the likelihood function, as shown in Eq. (2).


(2) $$\begin{array}{l} P(B_{1}=B_{1}^{i},B_{2}=B_{2}^{i}|I_{1},I_{2})=\frac{P(I_{1},I_{2}|B_{1}^{i},B_{2}^{i})\cdot P(B_{1}^{i},B_{2}^{i})}{\int P(I_{1},I_{2}|B_{1},B_{2})\cdot P(B_{1},B_{2})\text{d}B_{1},B_{2}}\\ \;\approx \frac{P(I_{1},I_{2}|B_{1}^{i},B_{2}^{i})}{\sum_{i}P(I_{1},I_{2}|B_{1}^{i},B_{2}^{i})}. \end{array}$$

Then, for the neural implementation of posterior probability, Shi and Griffiths (2009) have shown that divisive normalization $E\left(r_{i}/\underset{i}{\sum }r_{i}\right)$ is commonly found in the cerebral cortex by neuroscience experiments, and Eq. (3) has been proved, where *r*_*i*_ is the firing rate of the *i*^*th*^ neuron.


(3) $$E(r_{i}/\sum_{i}r_{i})=\frac{P(I_{1},I_{2}|B_{1}^{i},B_{2}^{i})}{\sum_{i}P(I_{1},I_{2}|B_{1}^{i},B_{2}^{i})}.$$

Next, we will describe the processes and mechanisms of probabilistic inference implemented in the neural network (adapted from Fang et al. 2019). First, for the process of probabilistic inference, the neural network processes external stimulus inputs *I*_1_ and *I*_2_ together in a bottom-up manner, as shown in Figure 3B. Second for the process of generation, which is to generate sampling neurons and the opposite of the inference process. Based on the generative model in Figure 3A, we can get sampling neurons $B_{1}^{i}$ and $B_{2}^{i}$ from *P*(*B*_1_) and *P*(*B*_2_), respectively. In other words, we can get that the sampling neurons follow $B_{1},B_{2}\sim N\left(0,\sigma^{2}\right)$.

### 3.2 Difficulties in designing the accelerator

In this work, the communication settings between PS and PL should be considered first in the design of the accelerator. Since the design requires frequent data interactions during operation, the selection of a suitable data interface can ensure the stability of data transmission while improving the time required for data transmission. The second is the design in the PL part, the design of this part is mainly to complete the work of the FPGA, which usually needs to achieve the purpose of acceleration by reducing the Latency of the design.

**Figure 4:**
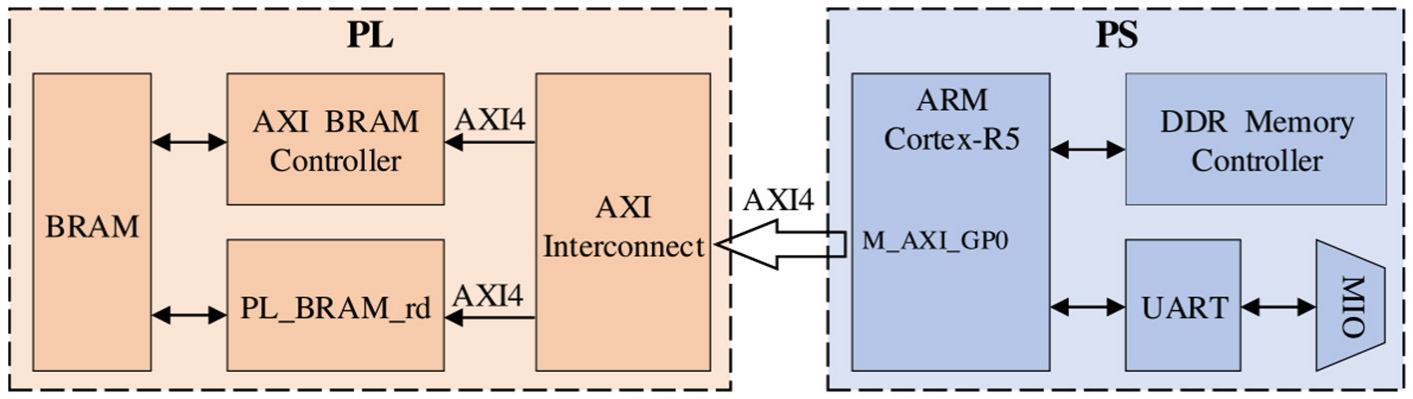
Data interaction architecture between PS and PL, here we use m_axi interface for data transmission.

For the communication setting between PS and PL, since the BRAM in PL part is not enough to store a large amount of data and parameters, it is necessary to exchange data frequently between the PL and PS parts. Therefore, in order to achieve high-speed read/write operations for large-scale data, we use the m_axi interface to realize it. Figure 4 shows the data interaction architecture between PS and PL. The m_axi interface has independent read-and-write channels, supports burst transfer mode, and the potential performance can reach 17GB/s, which fully meets our data scale and transfer speed requirements.

Furthermore, for the design of the PL part, since each node in the model contains a large number of neurons, it will take up a lot of resources, and clocks in the process of encoding, summing, multiplying, and normalizing neurons, in which loops may also be nested. Although pipelines can be added to the loops to improve the parallelism of the model operation, the optimization is not satisfactory due to the large number of bases. Therefore, we propose a highly parallelized structure by introducing an array division method to divide the array into blocks, which can further unroll the loop and make each loop execute independently to improve the degree of program parallelization. In short, it is a method of exchanging space for time.

## 4 Software and hardware optimizations

**Figure 5:**
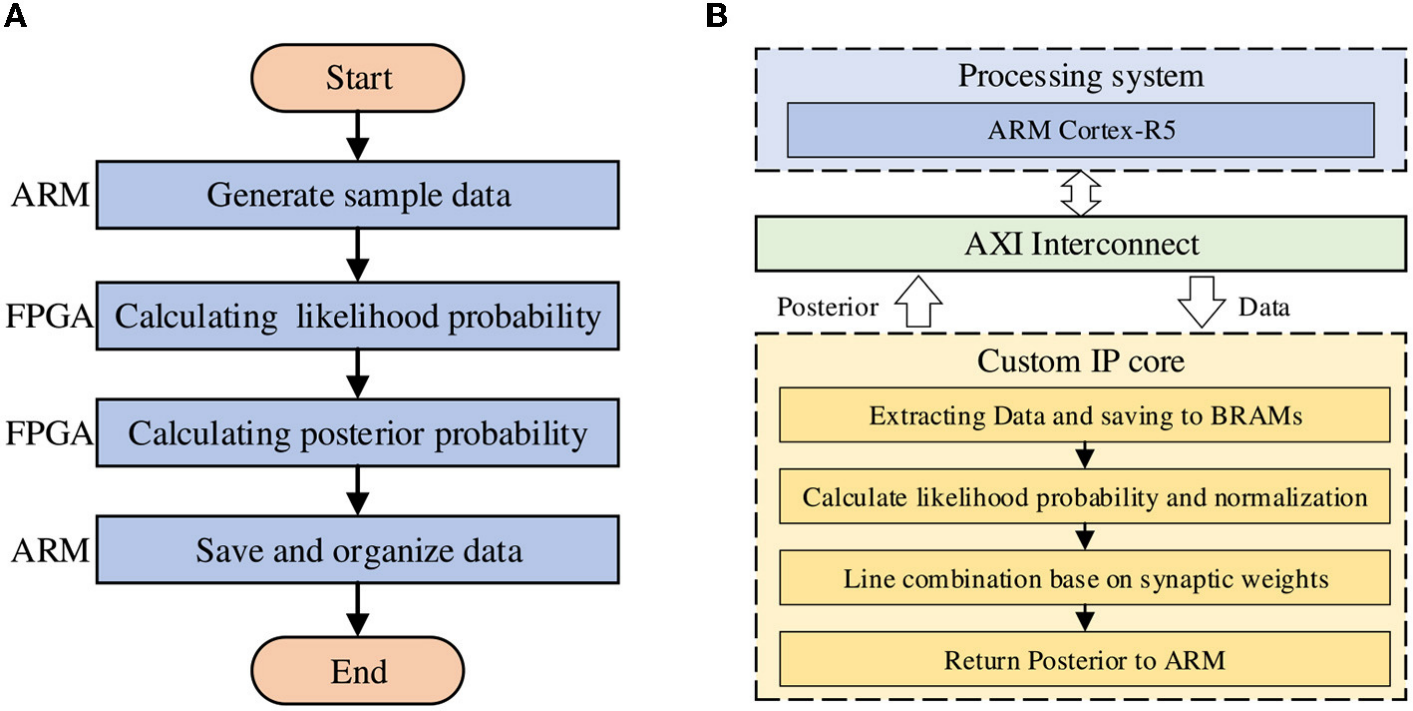
The design idea and overall computing architecture. **(A)** The program flow of the model on the ZCU104 board. **(B)** The hardware architecture of the model.

The design idea and overall architecture of this work are shown in Figure 5, which consists of ARM, AXI interface, and custom IP core designed by Vivado HLS. In the IP core part, we mainly use the structure of the streaming pipeline to reduce Latency and thus improve the operation speed. As mentioned in the previous section, we use the AXI master interface provided by Xilinx for data transmission between PS and PL, and the prior distribution and sample data that are ready to participate in inference will be allocated and stored in the on-chip BRAM. When the operation is finished, the result will also be returned to the off-chip DDR memory through the AXI master interface for subsequent processing.

In our work, we use the Vivado HLS tool provided by Xilinx to complete the design of the hardware IP core. This tool allows the synthesis of digital hardware directly using the high-level description developed in C/C++. With this tool we can convert C/C++ designs into RTL implementations for deployment on the FPGA, thereby significantly reducing the time required for FPGA development using traditional RTL descriptions. Therefore, the hardware architecture of the STM accelerator is designed by the programming language C++.

### 4.1 IP-core optimization

As mentioned in the last section, while adding the PIPELINE directive to the loop, we also use the method of array division to further improve the parallelism of the operation.

**Figure 6:**
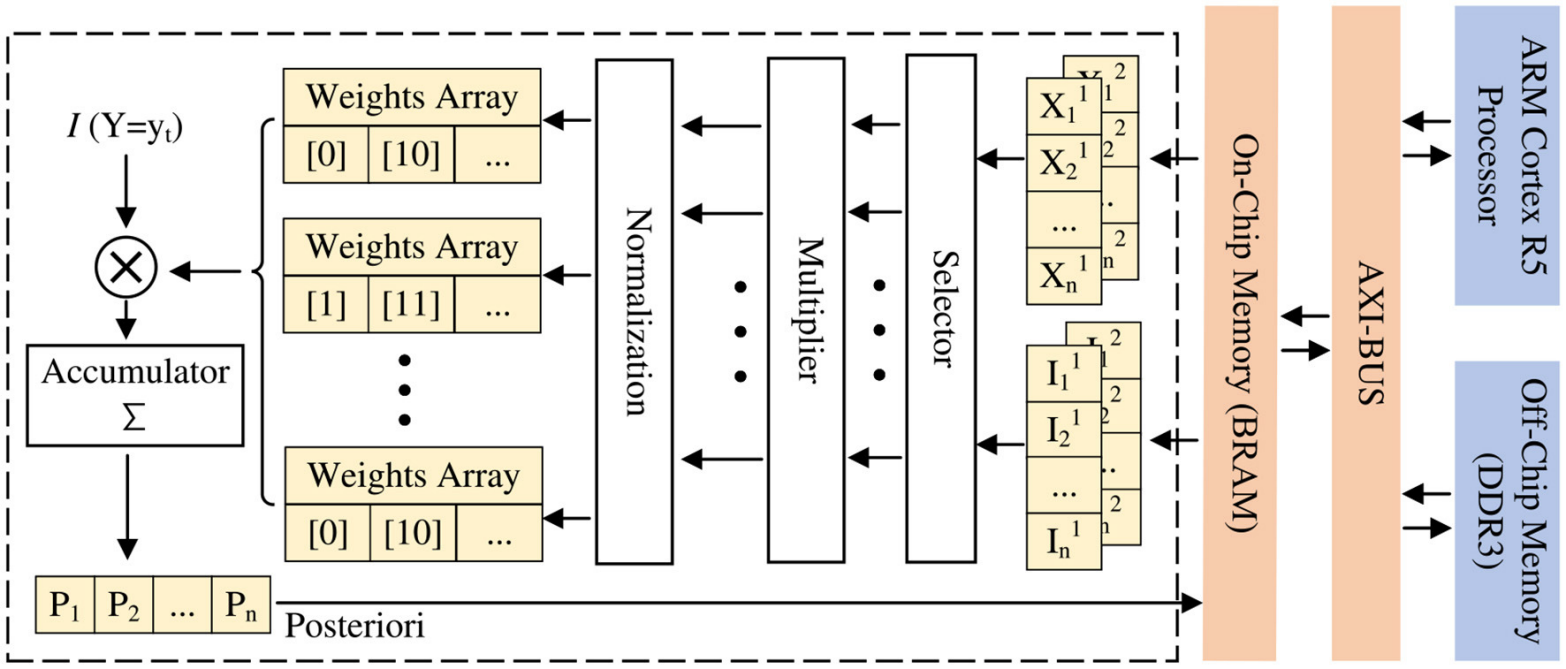
Design optimization ideas consisting of on-chip BRAM and processing elements (PE) using array division.

Here we take the sum of arrays as an example to illustrate how to improve parallelism. Under normal circumstances, the summation of an array is to iterate through each element of the array and accumulate them in turn. But even if we use the pipeline structure here, the accumulated value needs to be continuously read and written during the accumulation process. To prevent the emergence of dirty data, which leads to a time gap between the two loops, thus slowing down the speed of operation. In contrast, after we divide the original large-scale array into 10 blocks through array division, the subscripts of the array elements are accumulated every 10. In this way, the two adjacent loops in the accumulation process do not read and write to the same memory, thereby eliminating the time interval that would normally occur, to achieve the degree of parallelization of accumulation, as shown in Figure 6.

Finally, adding all blocks is the result of the array summation. The purpose of the manual expansion is to avoid memory access bottlenecks and increase the degree of parallelism while using DSP as much as possible. Table 1 is based on the Bayesian network model shown in Figure 3A. In the case of setting 1,000 neurons in each node, the resource consumption and latency of not using array segmentation and using array segmentation are compared. It can be seen that the resource consumption increases slightly with array segmentation, but the Latency decreases significantly.

**Table 1:** Comparison of resource consumption and Latency between the normal and the case using array division.

	**BRAM**	**DSP**	**FF**	**LUT**	**Latency**
Normal	14	172	25,934	38,817	11,170
Array division	14	179	28,142	43,849	6,698

In addition, to further reduce resource utilization and improve performance, we use a bit-width of 32 bits for each operation through a simple quantization of floating-point operations. This kind of quantization has a relatively low negative impact on accuracy and can improve the performance of each IP core without reducing the parameters and input accuracy. At the same time, to alleviate the problem of the maximum frequency increase caused by reusing the same hardware components, especially BRAM resources, we added input and output registers to each BRAM instance to meet the 10 ns clock cycle of each IP core. Algorithm 1 shows the pseudocode of the IP core design. By default, all nested loops are executed sequentially. During this process, Vivado HLS provides different pragmas to affect scheduling and resource allocation.

**Algorithm 1:**
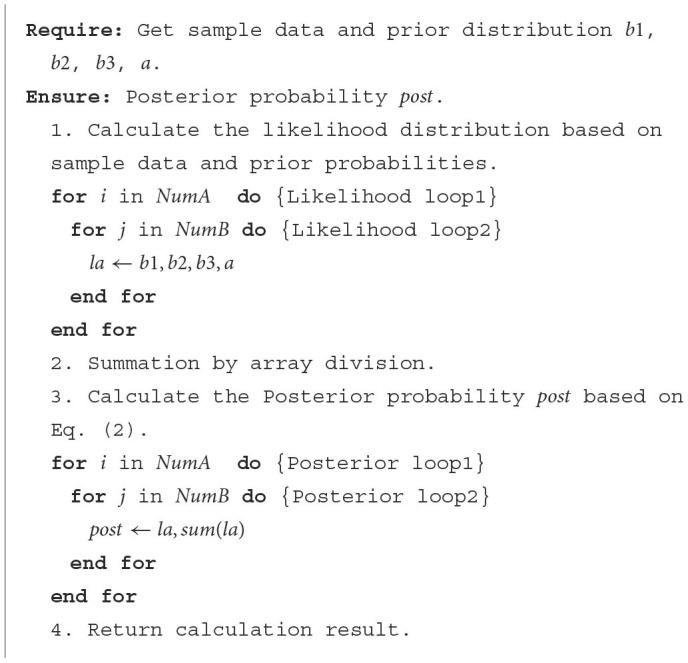
IP-core design in pseudo-code.

### 4.2 Interface signal control

When we compile the PL-side custom core, we need to set up the top-level file containing the form parameters and return values. These parameters are mapped to the hardware circuitry to generate interface signals, which can be controlled to not only help set better constraints but also to better control the input and output data flow according to the port timing. In addition, control logic needs to be extracted to form a state machine, so some handshake signals such as ap_start and ap_done will be formed.

**Figure 7:**
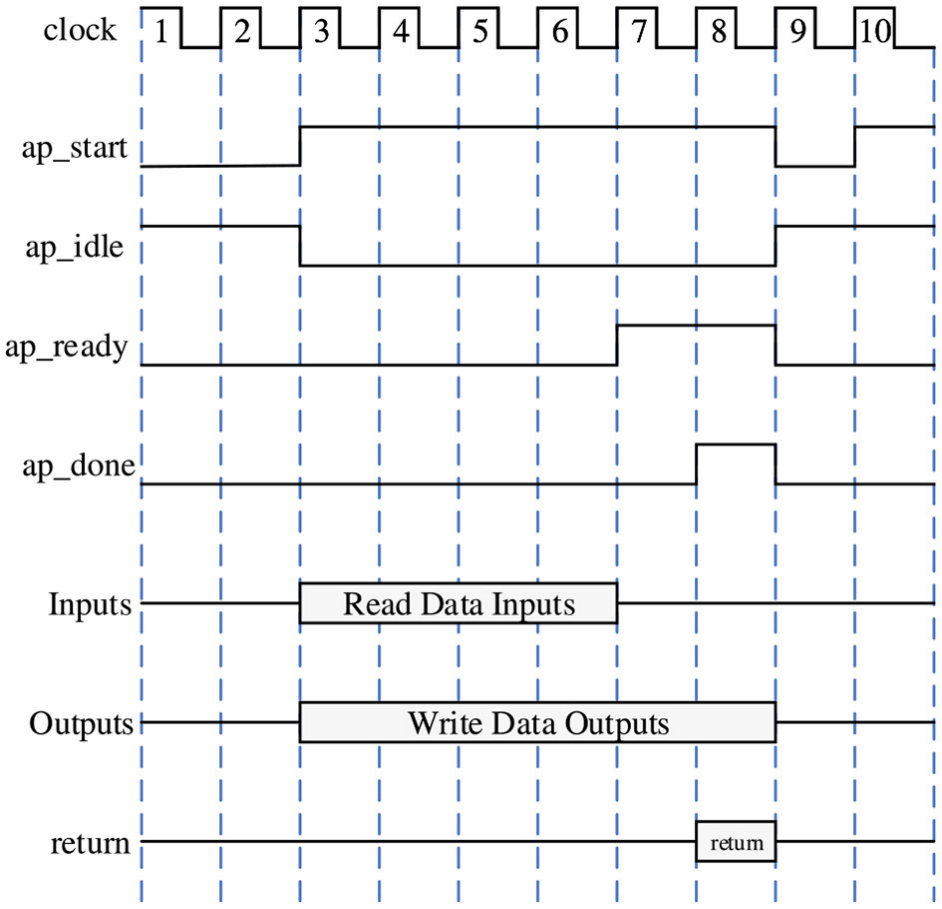
Timing diagram of ap_ctrl_hs four handshake signal functions. We mainly use ap_start interface to send read data commands to the FPGA, and detect ap_dong interface in real-time to determine whether the FPGA has completed the work.

Common interface constraints can be divided into Block-Level Protocols and Port-Level Protocols. Here we mainly use the ap_ctrl_hs signal in Block-Level Protocols, which contains four handshake signals ap_start, ap_idle, ap_ready, and ap_done. The ap_start signal is active high and indicates when the design starts working. The ap_idle signal is active low and indicates whether the design is idle. The ap_ready signal indicates whether the design is currently ready to receive new inputs. The ap_done signal indicates when the data on the output signal line is valid. The specific functional timing diagram is shown in Figure 7.

According to the timing diagram, we only need to pull the ap_start signal high and the design will automatically read or write data through the AXI bus while performing the inference operation. When the ap_done signal is read high, it means that the design has been completed, and the valid operation result can be obtained by reading the memory allocated for return.

### 4.3 Hardware–software streaming architecture

**Figure 8:**
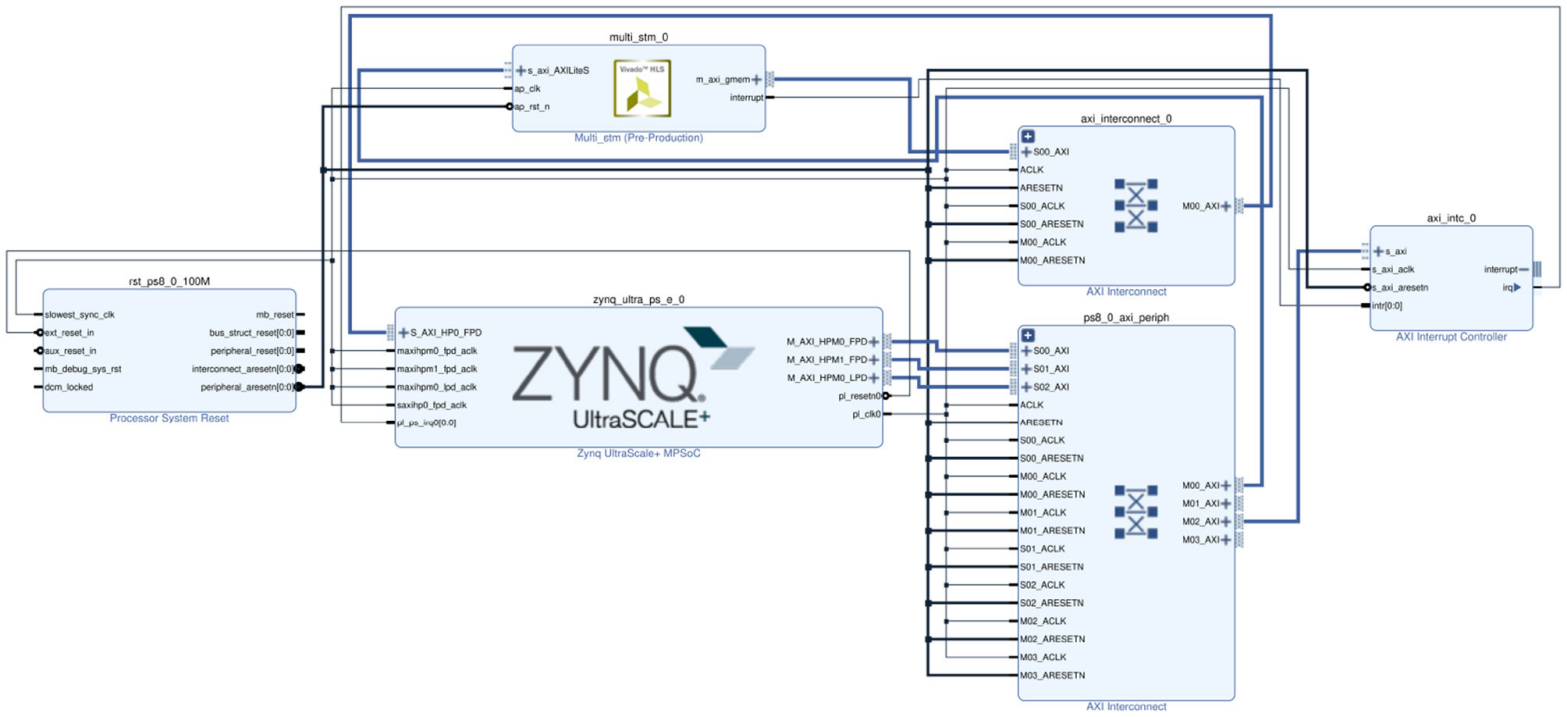
Hardware streaming architecture block design targeting the Soc with the m_axi interface between the PL and PS.

After the IP core has been designed, it is added to the Zynq block design to create the complete hardware architecture, as shown in Figure 8. The axi_interconnection module ensures communication between the IP core, PS system, and AXI interface. The axi_intc module controls the communication interruption of the interface.

Following the initialization of the design, the PS part will be used to implement the bitstream loading of the SNN. It also allows the PS to pass the values of external stimuli and SNN synaptic strengths to the PL part at runtime, which implements the specific neural network model. The main interface is used to connect the PL and PS parts of the SoC to ensure high-performance communication and data exchange between the IP-core and the PS in the streaming architecture. At the same time, the interlayer pipeline inside each IP-core is highly customized to build a Co-design with reset and GPIO. Both external stimulus values and synaptic strength values are stored in the cache of the BRAM in the PL part to improve the data reading speed for STM inference.

## 5 Simulations

We use the Intel i7-10700 and i5-12500, two of the more capable CPUs currently available, as benchmarks to compare the performance of model inference implemented on FPGAs. We test the performance and accuracy of the STM on the FPGA board for Bayesian inference on two brain perception problems: causal inference and multisensory integration. The evaluation metrics include inference effectiveness and processing speed on the model. In terms of inference effectiveness, causal inference is evaluated by the error rate varies with sample size, and multisensory integration is evaluated by comparison of the inference results and theoretical value.

### 5.1 Causal inference

Causal inference is the process by which the brain infers the causal effect between cause and outcomes when it receives external information (Shams and Beierholm, 2010). The core problem of causal inference is to calculate the probability of the cause, which can be expressed as the expectation value defined on the posterior distribution. The calculation of the posterior probability is converted into the calculation of the prior probability and the likelihood probability through importance sampling, to realize the simulation of the causal inference process in the brain. In this experiment, we verify the accuracy and efficiency of Bayesian inference in the STM on the Xilinx ZCU104 FPGA board because probabilistic sampling on SNNs involves a large number of probabilistic calculations that can consume a lot of time, and the processing of the data in the inference process involves many computation-intensive operations, and the CPU is not able to handle these tasks very quickly.

**Figure 9:**
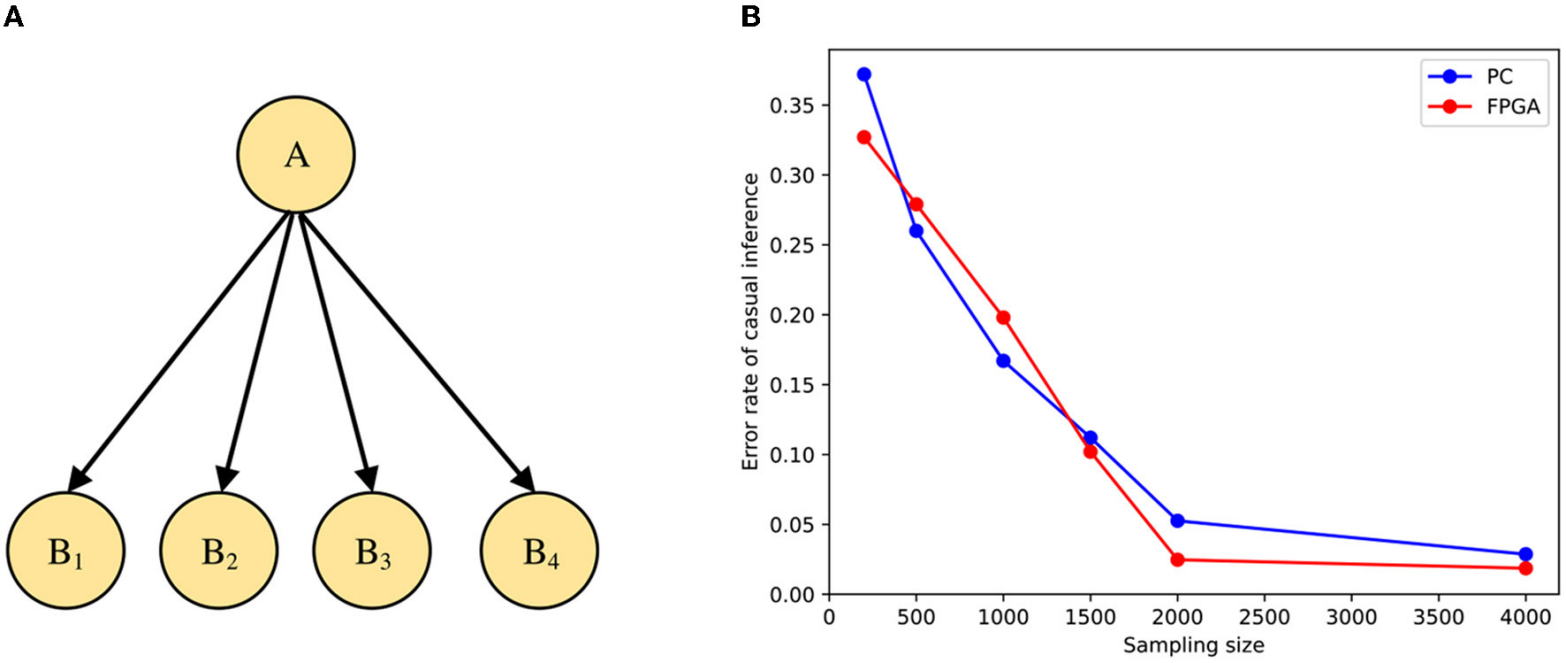
Simulation of causal inference. **(A)** The neural network architecture of the basic Bayesian network. **(B)** Comparison of error rates under PC and FPGA platforms.

In this paper, the validity of the model is verified from the accuracy of inference when different samples are input, and the STM is modeled by the Bayesian network shown in Figure 9A. Where *B*_1_, *B*_2_, *B*_3_, and *B*_4_ represent the input stimulus in causal inference and *A* denotes the cause. The tuning curve of each spiking neuron can be represented as the state of the variable. We suppose that the prior and conditional distributions are known, the distributions of these spiking neurons follow the prior distribution *P*(*B*_1_, *B*_2_, *B*_3_, *B*_4_), and the tuning curve of the neuron *i* is proportional to the likelihood distribution $P\left(B_{1}^{i},B_{2}^{i},B_{3}^{i},B_{4}^{i}|A\right)$. We can then normalize the output of Poisson spiking neurons through shunt inhibition and synaptic inhibition. Here we use *y*_*i*_ to denote the individual firing rate of the spiking neuron *i* and *Y* to denote the overall firing rate, and then:


(4) $$E(y_{i}/Y=n)=\frac{P(B_{1}^{i},B_{2}^{i},B_{3}^{i},B_{4}^{i}|A)}{\sum_{i}P(B_{1}^{i},B_{2}^{i},B_{3}^{i},B_{4}^{i}|A)}.$$

By multiplying and linearly combining the normalized results with the synaptic weights, the posterior probability can be calculated:


(5) $$P(A=a|B_{1},B_{2},B_{3},B_{4})=\sum_{l}I(A^{l}=a)\sum_{i}\frac{P(B_{1}^{i},B_{2}^{i},B_{3}^{i},B_{4}^{i}|A^{l})}{\sum_{l}P(B_{1}^{i},B_{2}^{i},B_{3}^{i},B_{4}^{i}|A^{l})}.$$

The results of the accuracy test are shown in Figure 9B. The error rate of the stimulus estimation keeps decreasing as the sample size increases, and when there are 2,000 sampled neurons, the error rate of stimulus estimation is already quite small. In addition, the inference accuracy of the implementation on the FPGA is similar to that on the PC. Therefore, the STM we run on the FPGA board can guarantee the accuracy of inference.

In terms of performance, we compare the design with multithreading and multiprogramming implementations on traditional computing platforms, and the results are shown in Table 2. It shows the processing time for each neuron sampling when the number of sampled neurons is 4,000. It can be seen from the results that multithreading and multiprogramming do not achieve the desired speedup but have the opposite effect. The possible reasons for this situation have been analyzed as follows: (1) Multithreaded execution is not strictly parallel, and global interpreter locks (GILs) can prevent parallel execution of multiple threads, so it may not be possible to take full advantage of multicore CPUs; (2) In terms of multiprogramming, perhaps the problem did not reach a certain size, resulting in the process creation process taking longer than the runtime. In addition, communication between multiprocesses requires passing a large amount of sample data, which introduces some overhead. For the above reasons, we finally considered using vectorization operations to vectorize the sample data to reduce the number of loops and avoid the speed limitations caused by nested loops.

**Table 2:** Results of sampling time and speed-up of each neuron in the two-layer model.

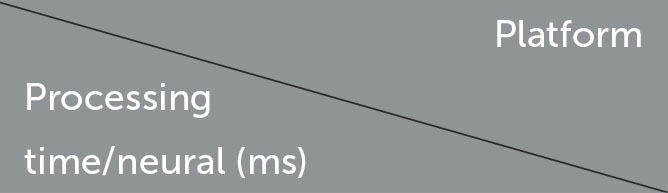		**Intel i7-10700** **2.90 GHz**	**Intel i5-12500** **2.50 GHz**	**ARM**	**Xilinx** **ZCU104**
Normal		8.556	4.315	53.814	**0.389**
Multithreading	2	12.217	6.091		
	4	13.098	6.907		
	10	13.355	7.578		
	20	13.778	8.386		
	50	16.085	10.631		
	100	20.323	14.772		
Multiprogramming	2	344.00	250.88		
	4	394.43	278.46		
	8	564.03	454.81		
	16	948.47	844.73		
Vectorization		**3.662**	**2.993**		

Bold values represents the optimal time on the corresponding platform.

From Table, we can see that vectorization is significantly faster than serial execution, multithreading, and multiprogramming, while the processing speed of the model on the FPGA is significantly better than that of the PC.

### 5.2 Causal inference with multi-layer neural network

**Figure 10:**
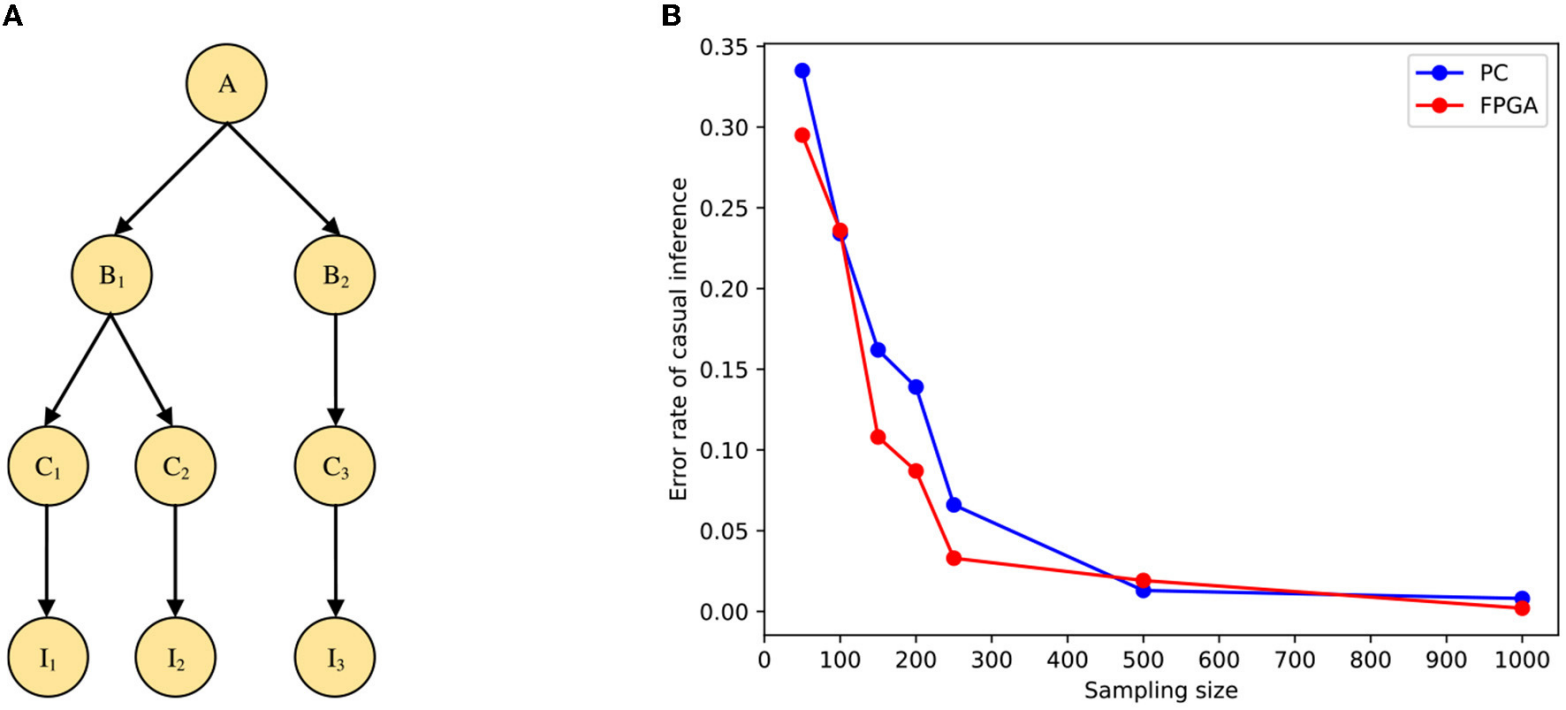
Simulation of causal inference with a multi-layer neural network. **(A)** The Bayesian model for multi-layer network structure. **(B)** Comparison of error rates under PC and FPGA platforms.

The simulation in the previous section verified the causal inference under a simple model. The inference speed on the CPU decreases exponentially as the problem size increases when the need to shorten the inference time on the network model through improvements and optimizations becomes even more important. In this section, we will use a multi-layer neural network model to test large-scale Bayesian inference based on the sampling tree on the FPGA board. The STM is modeled by the Bayesian network shown in Figure 10A, where *I*_1_, *I*_2_ and *I*_3_ denote the input stimuli in causal inference, *A* denotes the cause, and the rest are intermediate variables.

In this simulation, we use several spiking neurons to encode variables *C*_1_, *C*_2_, and *C*_3_ respectively, and the distribution of these neurons follows the prior distribution *P*(*C*_1_, *C*_2_) and *P*(*C*_3_). In addition, the tuning curves of these neurons are proportional to the distribution $P\left(I_{1},I_{2}|C_{1}^{i},C_{2}^{i}\right)$ and $P\left(I_{3}|C_{3}^{j}\right)$. We can obtain the average firing rates of spiking neurons $C_{1}^{i}$, $C_{2}^{i}$, and $C_{3}^{j}$, respectively:


(6) $$E(C_{1}^{i},C_{2}^{i})=\frac{P(I_{1},I_{2}|C_{1}^{i},C_{2}^{i})}{\sum_{i}P(I_{1},I_{2}|C_{1}^{i},C_{2}^{i})},$$

(7) $$E(C_{3}^{j})=\frac{P(I_{3}|C_{3}^{j})}{\sum_{j}P(I_{3}|C_{3}^{j})}.$$

The firing rate calculation of neurons in other layers is similar to this layer. The firing rate of each layer is multiplied and fed back to the next layer in the form of synaptic weights, and then the posterior probability can be calculated:


(8) $$\begin{array}{l} P(A=a|I_{1},I_{2},I_{3})=\\ \sum_{l}I(A^{l}=a)\sum_{k}\frac{P(B_{1}^{k},B_{2}^{k}|A^{l})}{\sum_{l}P(B_{1}^{k},B_{2}^{k}|A^{l})}\sum_{i,j}\frac{P(C_{1}^{i},C_{2}^{i},C_{3}^{j}|B_{1}^{k},B_{2}^{k})}{\sum_{k}P(C_{1}^{i},C_{2}^{i},C_{3}^{j}|B_{1}^{k},B_{2}^{k})}\\ \frac{P(I_{1},I_{2}|C_{1}^{i},C_{2}^{i})P(I_{3}|C_{3}^{j})}{\sum_{i}P(I_{1},I_{2}|C_{1}^{i},C_{2}^{i})\sum_{j}P(I_{3}|C_{3}^{j})} \end{array}$$

Similar to the simple model, the result of the STM under the multi-layer neural network on the FPGA is shown in Figure 10B. From the figure, we can see that the model running on the FPGA can guarantee the accuracy of the inference. Moreover, the performance comparison is shown in Table 3, in the multilayer network model, multithreading and multiprogramming are equally limited to achieve the desired results, so the same vectorization operation is used to optimize the program. We can also see the processing speed of the STM on FPGA is also improved compared with the traditional computing platform. In addition, we can find that due to the increase in the problem size of the multi-layer model, the acceleration of the model implemented on FPGA is more pronounced than in the two-layer model, even more than doubling.

**Table 3:** Results of sampling time and speed-up of each neuron in the multi-layer model.

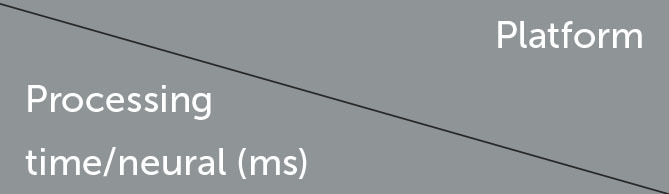		**Intel i7-10700** **2.90 GHz**	**Intel i5-12500** **2.50 GHz**	**ARM**	**Xilinx** **ZCU104**
Normal		1.103	0.635	12.75	**0.024**
Multithreading	2	1.048	0.622		
	4	1.019	0.617		
	10	1.006	0.617		
	20	1.012	0.618		
	50	1.012	0.618		
	100	1.013	0.624		
Multiprogramming	2	1.174	0.749		
	4	1.056	0.706		
	8	1.113	0.762		
	16	1.371	1.097		
Vectorization		**0.569**	**0.403**		

Bold values represents the optimal time on the corresponding platform.

### 5.3 Multisensory integration

In our daily life, we will obtain information from the outside world from the sense such as vision, hearing, and tough simultaneously, and the human brain can integrate this sensory information in the optimal way to get detailed information about an external object (Wozny et al., 2008). Some experiments have proved that the linear combination of different neuronal population activities with probabilistic population coding corresponds to the process of multisensory integration (Ma et al., 2006). Here, to demonstrate that our design can be generalized to other cognitive problems, we show that the STM on the FPGA board can solve multisensory integration problems with high performance and accuracy, and the final results can demonstrate that this work achieves good performance in the multisensory integration problem as well.

**Figure 11:**
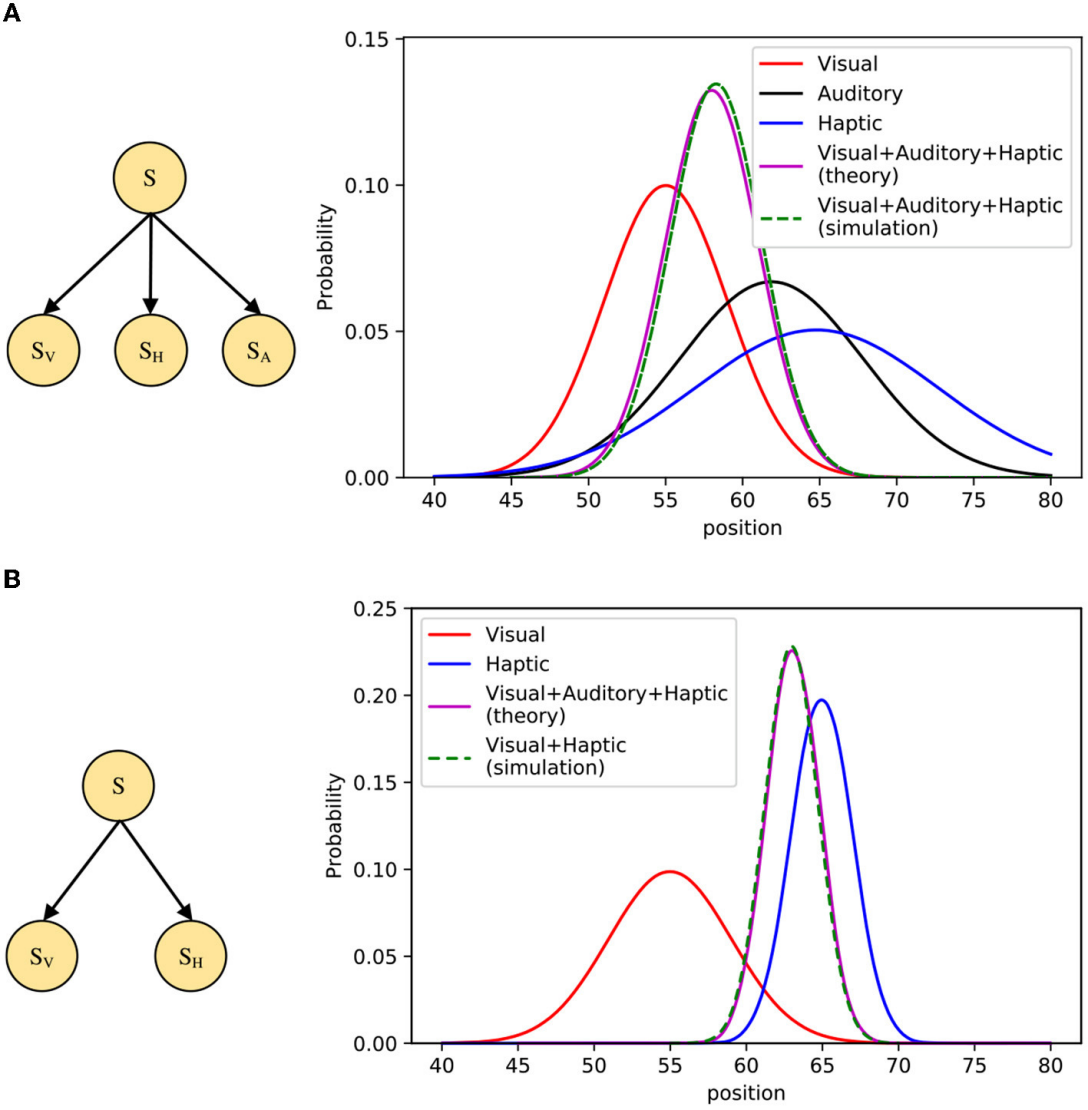
Simulation of multisensory integration. **(A)** Left: The Bayesian model for visual-auditory-haptic integration, Right: Comparison of model inference results and theoretical values on FPGA. **(B)** Left: The Bayesian model for visual-haptic integration, Right: Comparison of model inference results and theoretical values on FPGA.

The simulation first considers the visual-auditory-haptic integration problem, and the STM is modeled by the Bayesian network shown in Figure 11A. Here *S* denotes the position of the object stimulus, *S*_*V*_, *S*_*H*_, and *S*_*A*_ denote visual, auditory, and haptic cues, respectively. We suppose that *P*(*S*) is a uniform distribution, *P*(*S*_*V*_|*S*), *P*(*S*_*H*_|*S*), and *P*(*S*_*A*_|*S*) are three Gaussian distributions, respectively. When given *S*_*V*_, *S*_*H*_, and *S*_*A*_, we can use importance sampling to infer the posterior probability of *S*, as:


(9) $$\begin{array}{l} P(S=s|S_{V},S_{H},S_{A})=\sum_{S}I(S=s)P(S|S_{V},S_{H},S_{A})\\ \;=\sum iI(S_{i}=s)\frac{P(S_{V},S_{H},S_{A}|S_{i})}{\sum iP(S_{V},S_{H},S_{A}|S_{i})}\;,\;S_{i}\sim P(s). \end{array}$$

In our simulation, multisensory integration inference is achieved through neural circuits based on PPC and normalization. We use 1,000 spiking neurons to encode stimuli whose states follow the prior distribution *P*(*S*). We suppose that the tuning curve of the neuron *i* is proportional to the distribution *P*(*S*_*V*_, *S*_*H*_, *S*_*A*_|*S*_*i*_), and then use shunting inhibition and synaptic depression to make the output of spiking neurons normalized, the result will be fed into the next spiking neuron with synaptic weights *I*(*S*_*i*_ = *s*). Figure 11A shows the simulation results, where the inference result obtained from the STM on the FPGA board is in good agreement with the theoretical values. Similar to the visual-auditory-haptic integration, we also add a simulation of visual-haptic integration to improve the completeness, which is illustrated in Figure 11B. Furthermore, the performance comparison is shown in Table 4, which shows a significant improvement in the sampling speed of each neuron on the FPGA. Since the results of multi-threading and multi-process experiments were not ideal in previous experiments, only vectorization methods are compared here. The results also show that the running speed on FPGA is still better than that on CPU.

**Table 4:** Results of sampling time and speed-up of each neuron in the simulation of multisensory integration.

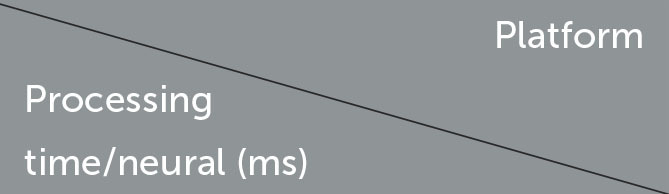	**Intel i7-10700** **2.90 GHz**	**Intel i5-12500** **2.50 GHz**	**ARM**	**Xilinx** **ZCU104**
Normal	7.632	5.169	94.608	**0.328**
Vectorization	**3.882**	**2.160**		

Bold values represents the optimal time on the corresponding platform.

## 6 Conclusion

In this work, we design an FPGA-based hardware accelerator for PGM-based SNNs with the help of the PYNQ framework. Firstly, the STM, as a novel SNN simulation model for causal inference, can convert a global complex inference problem into a local simple inference problem, thus realizing high-precision approximate inference. Furthermore, as a generalized neural network model, the STM does not formulate a neural network for a specific task and thus can be generalized to other problems. Our hardware implementation is based on this solid and innovative theoretical model, which solves the problem of slow model computation based on its realization of large-scale multi-layer complex model inference.

Secondly, As the first work to realize the hardware acceleration of the STM, we chose the FPGA platform as the acceleration platform of the model. For CPUs and GPUs, both of them need to go through operations such as fetching instructions, decoding, and various branch logic jumps, and the energy consumption of GPUs is too high. In contrast, the function of each logic unit of an FPGA is determined at the time of reprogramming and does not require these instruction operations, so FPGAs can enjoy lower latency and energy consumption. Compared to hardware platform ASICs, FPGAs are more flexible. Although ASICs are superior to FPGAs in terms of throughput, latency, and power consumption, their high cost and long cycle time cannot be ignored, and the design of an ASIC cannot be easily changed once it is completed. In contrast, FPGAs are programmable hardware that can be changed at any time according to demand without having to remanufacture the hardware, and this flexibility is the reason why we ultimately chose FPGAs. FPGA is a compromise between the above two platforms, although some aspects of the performance is not up to the two, but it is a combination of the advantages of the two. It also provides reasonable cost, low power consumption, and reconfigurability for neuromorphic computing acceleration.

Thirdly, The experimental results and data on causal inference validate our conclusion: in the two-layer model, we can then see that the inference accuracy of the implementation on the FPGA can approximate that of the implementation on the CPU, with an accuracy of up to 98%, and at the same time achieve a multifold speedup. The acceleration effect becomes more and more obvious as the problem size increases, which is proved in the multi-layer model, and from the results we can see that the acceleration effect in the multi-layer model is more than twice as much as that in the two-layer model. Moreover, in the experiments on multisensory integration, the experimental results also demonstrate that our design implementation can be used for other real-world cognitive problems while guaranteeing the accuracy of reasoning and the acceleration effect.

Finally, the hardware acceleration method proposed in the paper can simulate the working principle of biological neurons very well. Meanwhile, due to the characteristics of low power consumption and real-time response of FPGA, this method can have a wide range of applications in the embedded field. The realized causal inference problems can be used in policy evaluation, financial decision-making and other fields, and the multisensory integration can be used in vehicle environment perception, medical diagnosis and other fields. Specifically, in application scenarios such as smart home application environments, causal inference can be used to achieve reasoning about factors affecting health and provide personalized health advice. Sensory cues such as vision and hearing are combined to provide a better perceive the home environment and thus provide intelligent control. Our work provides a solution for such application scenarios and these practical applications are expected to promote the progress of the neuromorphic computing field and make it better meet the practical application requirements. In addition, so far the STM does not consider learning, which is an important aspect of adaptation between tasks. All the results of our simulations are based on inference with known prior probabilities and conditional probabilities. Therefore, in future work, we need to combine learning and inference into one framework and introduce some learning mechanisms to make the model more complete and flexible for multiple tasks.

## Data availability statement

The raw data supporting the conclusions of this article will be made available by the authors, without undue reservation.

## Author contributions

HL: Methodology, Data curation, Investigation, Software, Validation, Writing – original draft. BW: Methodology, Conceptualization, Supervision, Writing – review & editing. QL: Methodology, Writing – review & editing, Project administration. YF: Methodology, Writing – review & editing, Conceptualization, Formal analysis, Software, Supervision, Writing – original draft. JL: Formal analysis, Supervision, Writing – review & editing, Project administration. LA: Supervision, Writing – review & editing, Conceptualization, Methodology.
